# Bacterial degradation of monocyclic aromatic amines

**DOI:** 10.3389/fmicb.2015.00820

**Published:** 2015-08-18

**Authors:** Pankaj K. Arora

**Affiliations:** School of Biotechnology, Yeungnam UniversityGyeongsan, South Korea

**Keywords:** aniline, anthranilic acid, aminophenols, biodegradation, bioremediation

## Abstract

Aromatic amines are an important group of industrial chemicals, which are widely used for manufacturing of dyes, pesticides, drugs, pigments, and other industrial products. These compounds have been considered highly toxic to human beings due to their carcinogenic nature. Three groups of aromatic amines have been recognized: monocyclic, polycyclic, and heterocyclic aromatic amines. Bacterial degradation of several monocyclic aromatic amines has been studied in a variety of bacteria, which utilizes monocyclic aromatic amines as their sole source of carbon and energy. Several degradation pathways have been proposed and the related enzymes and genes have also been characterized. Many reviews have been reviewed toxicity of monocyclic aromatic amines; however, there is lack of review on biodegradation of monocyclic aromatic amines. The aim of this review is to summarize bacterial degradation of monocyclic aromatic amines. This review will increase our current understanding of biochemical and molecular basis of bacterial degradation of monocyclic aromatic amines.

## Introduction

Aromatic amines are derivatives of aromatic hydrocarbons containing an amino group, (-NH_2_) or an amine group (-NH), or a nitrogen (-N) atom in their structures. There are three types of aromatic amines: monocyclic, polycyclic, and heterocyclic, which have been observed in tobacco smoke, diesel exhaust, dyes, pesticides, pharmaceuticals, and polyurethane foams (Stellman, 1998; DeBruin and Josephy, 2002; DeBruin et al., 2002).

Many aromatic amines are recognized as known or suspect human carcinogens, and mutagenicity of aromatic amines has been demonstrated in many test systems, including Big Blue transgenic mice (Layton et al., 1995; Suter et al., 1996; Stellman, 1998; Chung, 2000; DeBruin and Josephy, 2002; DeBruin et al., 2002; Pira et al., 2010). Furthermore, they are potent inducer of the formation of methemoglobinemia in animals and humans (Ohta et al., 1983). Occupational exposure to aromatic amines causes an increased risk of bladder cancer in workers even 30 years after exposure (Pira et al., 2010). Several pesticides including diuron, metobromuron, linuron, isoproturon, chlorotoluron, acetochlor, bentazon, butachlor, metolachlor, amitraz, and vinclozolin may release several monocyclic aromatic amines in soil because of their microbial transformation (Dupret et al., 2011). Cigarette smoke releases several carcinogenic aromatic amines including p-toluidine, 2-naphthylamine, and 4-aminobiphenyl into the ambient air (Stabbert et al., 2003). Bladder cancer is strongly associated with cigarette smoking, probably due to exposure to aromatic amines in tobacco smoke (Pfeifer et al., 2002). Heterocyclic aromatic amines are generally produced in meats or fish when grilled or cooked at high temperatures (Steck et al., 2007). An epidemiological study showed that people who lifelong consume grilled meats and fish have a risk of post-menopausal breast cancer (Steck et al., 2007). Furthermore, higher exposures to heterocyclic aromatic amines may cause presence of DNA adducts, which are associated with carcinogenesis (Turesky, 2007).

Several reviews have been published dealing with toxicity of aromatic amines (Chung et al., 1997; Skipper et al., 2010; Besaratinia and Tommasi, 2013). Despite the fact, monocyclic aromatic amines are distributed throughout the environment including soil and groundwater, there is no review dealing with bacterial degradation of monocyclic aromatic amines. The aim of this review is to summarize bacterial degradation of monocyclic aromatic amines.

## Bacterial Degradation of Monocyclic Aromatic Amines

Many bacteria have been isolated and characterized with their ability to mineralize or transform various monocyclic aromatic amines. **Table 1** summarizes the role of various monocyclic aromatic amine-degrading bacteria. Bacterial degradation of monocyclic aromatic amines proceeds generally with release of ammonia. Ammonium ions may release either after the ring cleavage (Takenaka et al., 1997) or prior to the ring cleavage (Chang et al., 2003). Several mechanisms have been proposed for mineralization of monocyclic aromatic amines. Bacterial aerobic degradation of monocyclic aromatic amines may be initiated via one of the following mechanisms: (i) A dioxygenase may catalyze ring cleavage of aromatic amine (Takenaka et al., 1997), (ii) Dioxygenation of aromatic amine (Chang et al., 2003), (iii) Deamination of aromatic amine (Qu and Spain, 2011), (iv) Hydroxylation of aromatic amine (Takenaka et al., 2003), (v) Co-ligase mediated activation of aromatic amines to coenzyme A (CoA) thioesters (Schühle et al., 2001), and (vi) Dehalogenation of chlorinated aromatic amine (Hongsawat and Vangnai, 2011). In this section, the bacterial degradation of well-studied monocyclic aromatic amines including aniline, aminophenols, chloroaminophenols, anthranilate, 5-nitroanthranilate, 4-amino-3-hydroxybenzoate, methylanilines, and chloroanilines are discussed.

**Table 1:** A list of bacteria involved in degradation of monocyclic aromatic amines.

Bacteria	Aromatic amine(s)	Mode of action	Reference
*Acinetobacter* sp. YAA	Aniline	Aerobic, degraded via catechol and its *meta*-cleavage pathway	Fujii et al. (1997)
*Delftia tsuruhatensis* AD9	Aniline	Aerobic, degraded via catechol and its *meta*-cleavage pathway	Liang et al. (2005)
*Delftia* sp. XYJ6	Aniline	Aerobic, degraded via catechol and its *ortho*-cleavage pathway	Chengbin et al. (2009)
*Delftia* sp. HY99	Aniline	Both aerobic and anaerobic, Aerobically degraded via catechol, and anaerobically transformed to 4-aminobenzoic acid	Kahng et al. (2000)
*Desulfobacterium aniline*	Aniline	Anaerobic, degraded via 4-aminobenzoic acid and 4-aminobenzoyl-CoA	Schnell and Schink (1991)
*Frateuria* sp. ANA-18	Aniline	Aerobic, degraded via catechol and its *ortho*-cleavage pathway	Murakami et al. (2003)
*Pseudomonas* sp. AW-2	Aniline	Aerobic, degraded via catechol and its *meta*-cleavage pathway	Murakami et al. (1998)
*Burkholderia xenovorans* LB400	2-Aminophenol	Aerobic, degraded via direct ring cleavage to 2-aminomuconic-6-semialdehyde	Chirino et al. (2013)
*Pseudomonas* sp. AP-3	2-Aminophenol	Aerobic, degraded via direct ring cleavage to 2-aminomuconic-6-semialdehyde	Takenaka et al. (2005)
*Pseudomonas pseudoalcaligenes* JS45	2-Aminophenol	Aerobic, degraded via direct ring cleavage to 2-aminomuconic-6-semialdehyde	Nishino and Spain (1993)
*Burkholderia* sp. AK-5	4-Aminophenol	Aerobic, degraded via 1,4-benzenediol and 1,2,4-benzenetriol	Takenaka et al. (2003)
*Arthrobacter* sp. SPG	2-Chloro-4-aminophenol	Aerobic, degraded via chlorohydroquinone and hydroquinone	Arora et al. (2014a)
*Burkholderia* sp. RKJ 800	4-Chloro-2-aminophenol	Aerobic, degraded via 4-chlorocatechol	Arora et al. (2014b)
*Acinetobacter* sp. ADP1	Anthranilate	Aerobic, degraded via the catechol pathway	Eby et al. (2001)
*Azoarcus evansii*	Anthranilate	Both aerobic and anaerobic, Aerobically degraded via 2-aminobenzoyl-CoA and anaerobically degraded via benzoyl CoA.	Schühle et al. (2001)
*Burkholderia cepacia* DBO1	Anthranilate	Aerobic, degraded via the catechol pathway	Chang et al. (2003)
*Geobacillus thermodenitrificans* NG80-2	Anthranilate	Aerobic, via 3-hydroxyanthranilate	Liu et al. (2010)
*Nocardia opaca*	Anthranilate	Aerobic, degraded via the catechol pathway and the gentisate pathway	Cain (1968)
*Pseudomonas aeruginosa* PAO1	Anthranilate	Aerobic, degraded via the catechol pathway	Costaglioli et al. (2012)
*Pseudomonas* sp. PAMC 2593	Anthranilate	Aerobic, degraded via the catechol pathway	Kim et al. (2015)
*Bradyrhizobium* sp. JS329	5-Nitroanthranilate	Aerobic, degraded via 5-nitrosalicylic acid	Qu and Spain (2010)
*Bordetella* sp. 10d	4-Amino-3-hydroxybenzoate	Aerobic, degraded via direct ring cleavage	Orii et al. (2006)
*Desulfobacula toluolica* Tol2	4-Methylaniline	Anaerobic, transformed into *p*-aminophenylacetic acid and phenylacetic acid	Raber et al. (1998)
*Pseudomonas testosterone*	4-Methylaniline	Aerobic, degraded via 4-methyl-catechol	Raabe et al. (1984)
*Pseudomonas cepacia* CMA1	3-Chloro-4-methylaniline	Aerobic, degraded via release of ammonium and chloride ions	Stockinger et al. (1992)
*Acinetobacter baylyi* GFJ2	3,4-Dichloroaniline	Aerobic, degraded via 4-chloroaniline	Hongsawat and Vangnai (2011)
*Alcaligenes faecalis* H1	3,4-Dichloroaniline	Aerobic, degraded via 4,5-dichloropyrocatechol	Surovtseva et al. (1993)
*Pseudomonas fluorescens* 26-*K*	3,4- Dichloroaniline	Aerobic, degraded via 4-amino-2-chlorophenol	Travkin et al. (2003)
*Rhodococcus rhodochrous* CTM	2-Methylaniline, 4-Chloro-2-methylaniline, and 3-Chloro-2-methylaniline	Aerobic, degraded via corresponding methylcatechols	Fuchs et al. (1991)
*Brevundimonas* diminuta INMI KS-7	3-Chloroaniline, 4-Chloroaniline, and 3,4-Dichloroaniline	Aerobic, degraded via corresponding chloropyrocatechols	Surovtseva et al. (1986)
*Diaphorobacter* PCA039	4-Chloroaniline	Aerobic, degraded via 4-chlorocatechol	Zhang et al. (2010b)
*Bacillus megaterium* IMT21	2,3-Dichloroaniline (2,3-DCA), 2,4-Dichloroaniline (2,4-DCA), 2,5-Dichloroaniline (2,5-DCA), 3,4-Dichloroaniline (3,4-DCA), and 3,5-Dichloroaniline (3,5-DCA)	Aerobic, 2,3-DCA, 2,4-DCA, and 2,5-DCA degraded via dichloroaminophenols whereas 3,4-DCA and 3,5-DCA degraded via dichloroacetanilides	Yao et al. (2011)

## Bacterial Degradation of Aniline

Aniline, which is the simplest aromatic amine, is mainly used for synthesis of dyes, antioxidants, rubbers, pharmaceuticals, and herbicides (Stellman, 1998). In this subsection, pathways for aerobic and anaerobic bacterial degradation of aniline are summarized. Several aerobic bacteria have been isolated and characterized for mineralization of aniline (Fujii et al., 1997; Fukumori and Saint, 1997; Murakami et al., 1998; Liang et al., 2005; Chengbin et al., 2009). These bacteria initiate aniline degradation with formation of catechol that is degraded further via the *ortho*-cleavage or the *meta*-cleavage pathway. *Pseudomonas putida* UCC22 (Fukumori and Saint, 1997), *Acinetobacter* sp. YAA (Fujii et al., 1997), *Pseudomonas* sp. AW-2 (Murakami et al., 1998), and *Delftia tsuruhatensis* AD9 (Liang et al., 2005) metabolize aniline via the *meta*-cleavage pathway whereas*, Frateuria* sp. ANA-18 (Murakami et al., 2003) and *Delftia* sp. XYJ6 (Chengbin et al., 2009) degrade aniline via the *ortho*-cleavage pathway.

**FIGURE 1:**
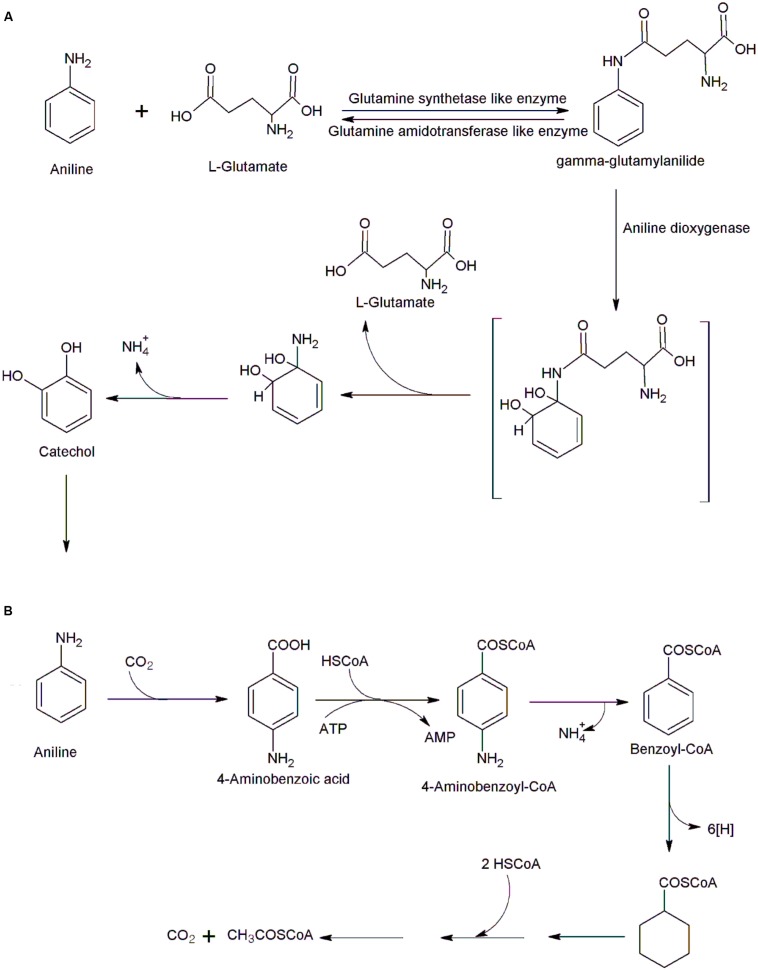
**Bacterial degradation of aniline. (A)** Initial steps of aniline degradation in *Acinetobacter* sp. YAA (Takeo et al., 2013), and **(B)** anaerobic degradation of aniline in *Desulfobacterium aniline* (Schnell and Schink, 1991).

The initial conversion of aniline to catechol is a multistep reaction catalyzed by three enzymes, a glutamine synthetase (GS)-like enzyme, glutamine amidotransferase like enzyme, and an aniline dioxygenase (a large and small subunits of an oxygenase component and a ferredoxin-reductase component; Fujii et al., 1997; Fukumori and Saint, 1997; Murakami et al., 1998; Liang et al., 2005). In the first step, GS like enzyme catalyzed ATP-dependent ligation of L-glutamate to aniline to form gamma-glutamylanilide (**Figure 1A**; Takeo et al., 2013). The next step, catalyzed by aniline dioxygenase involves conversion of gamma-glutamylanilide into catechol (Takeo et al., 2013). High concentrations of gamma-glutamylanilide are cytotoxic, but the action of another enzyme, glutamine amidotransferase, prevents its accumulation by converting it to aniline (Takeo et al., 2013). Five genes encoding these three enzymes involved in the conversion of aniline to catechol have been identified in a number of bacteria including *P. putida* UCC22 (Fukumori and Saint, 1997), *Acinetobacter* sp. YAA (Fujii et al., 1997), *Frateuria* sp. ANA-18 (Murakami et al., 2003), *Delftia acidovorans* 7N (Urata et al., 2004), *Delftia tsuruhatensis* AD9 (Liang et al., 2005) and *Delftia* sp. AN3 (Zhang et al., 2008). These genes are located on either plasmid or chromosomal DNA. The plasmids of *P. putida* UCC22 (pTDN1,) and *Acinetobacter* sp. YAA (pYA1) contain aniline oxidation genes (*tdnQTA1A2B* or *atdA1A2A3A4*; Fujii et al., 1997; Fukumori and Saint, 1997). Murakami et al. (2003) expressed all five genes from *Frateuria* sp. ANA-18 in *Escherichia coli* and the recombinant bacteria exhibited the aniline oxidation activities. They demonstrated that deletion of *tdnA1A2* or *tdnQ* genes resulted in loss of aniline oxidation activity. Apart of a *tdn* gene cluster, *Frateuria* sp. contain two catechol catabolic gene clusters *cat1* and *cat2* (Murakami et al., 2003). The gene cluster *cat1* may involve in the *ortho*-cleavage pathway of aniline degradation (Murakami et al., 2003). Takeo et al. (2013) reported characterization of the *atdA1* gene (encoding the enzyme similar to GS) from *Acinetobacter* sp. YAA and confirmed that the AtdA1 catalyzes conversion of aniline to gamma-glutamylanilide.

Anaerobic degradation of aniline was studied in sulfate-reducing bacterium *Desulfobacterium aniline* (Schnell and Schink, 1991). Initially, aniline is carboxylated to 4-aminobenzoic acid that is transformed to 4-aminobenzoyl-CoA (**Figure 1B**). The 4-aminobenzoyl-CoA undergoes reductive deamination to form benzoyl-CoA which enters the normal benzoate pathway, to form three acetyl-CoA. Few bacteria are able to degrade aniline under either aerobic or anaerobic conditions; *Delftia* sp. HY99 is one example of this capability (Kahng et al., 2000). Strain HY99 mineralized aniline acerbically via catechol and transformed aniline to 4-aminobenzoic acid under anaerobic conditions (Kahng et al., 2000).

## Bacterial Degradation of Aminophenols

**FIGURE 2:**
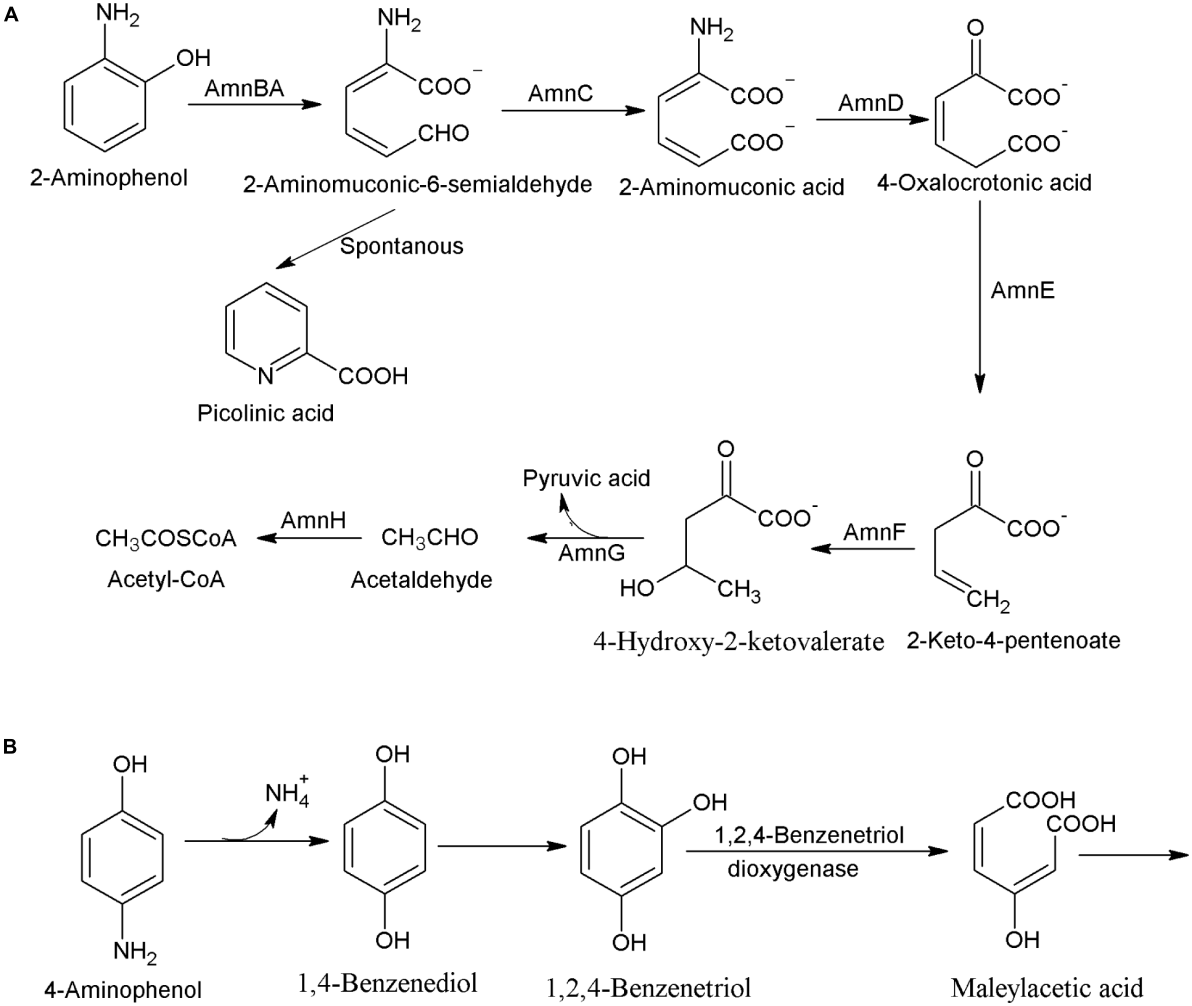
**Bacterial degradation pathway of **(A)** 2-aminophenol in *Pseudomonas* sp. AP3 (Murakami et al., 1998), and *Burkholderia xenovorans* LB400 (Chirino et al., 2013), and **(B)** 4-aminophenol in *Burkholderia* sp. AK-5 (Takenaka et al., 2003)**.

2-Aminophenol and 4-aminophenol are two major isomers of aminophenol, which are widely used for pharmaceuticals, drugs, and dyes. In this subsection, the pathways for bacterial degradation of 2-aminophenol and 4-aminophenol are summarized. The aerobic bacterial degradation of 2-aminophenol initiates with ring-cleavage of 2-aminophenol to 2-aminomuconic-6-semialdehyde by the 2-aminophenol-1,6-dioxygenase (AmnBA; Takenaka et al., 1997). The next step, catalyzed by 2-aminomuconic acid dehydrogenase (AmnC) involves conversion of 2-aminomuconic-6-semialdehyde to 2-aminomuconic acid that deaminates into 4-oxalocrotonic acid by the 2-aminomuconate deaminase (AmnD) with a concomitant release of ammonium (**Figure 2A**). In next step, 4-oxalocrotonic acid decarboxylase (AmnE) catalyzes conversion of 4-oxalocrotonic acid to 2-keto-4-pentenoate that is transformed to 4-hydroxy-2-ketovalerate by a hydratase (AmnF; Chirino et al., 2013). Next degradation step involves conversion of 4-hydroxy-2-ketovalerate to pyruvic acid and acetaldehyde by a 4-hydroxy-2-oxovalerate aldolase (AmnG; Chirino et al., 2013). Acetaldehyde is further converted to acetyl coenzyme by acetaldehyde dehydrogenase (AmnH). An *amn* gene cluster involved in the 2-aminophenol degradation has been observed in *Pseudomonas* sp. AP-3 (Takenaka et al., 2005), *P. pseudoalcaligenes* JS45 (Nishino and Spain, 1993), *P. putida* HS12 (Park and Kim, 2000), *P. knackmussi* B13 (Gaillard et al., 2006), and *Burkholderia xenovorans* LB400 (Chirino et al., 2013). In *Burkholderia xenovorans* LB400, the ring cleavage product, 2-aminomuconic-6-semialdehyde is partially converted to picolinic acid that reduced bacterial growth during the 2-aminophenol degradation (Chirino et al., 2013). Literature studies also show the non-enzymatic conversion of 2-aminomuconic-6-semialdehyde to picolinic acid in absence of nicotinamide adenine dinucleotide (Nishino and Spain, 1993).

The degradation pathway of 4-aminophenol was studied in *Burkholderia* sp. AK-5 that utilized it as its sole source of carbon, nitrogen, and energy (Takenaka et al., 2003). Initially, 4-aminophenol is hydroxylated to 1,4-benzenediol that is further hydroxylated to 1,2,4-benzenetriol (Takenaka et al., 2003). The next step, catalyzed by a 1,2,4-benzenetriol dioxygenase involves ring-cleavage of 1,2,4-benzenetriol to maleylacetic acid (**Figure 2B**). This bacterium expresses a Fe-containing superoxide dismutase and a 2-hydroxy-1,4-benzoquinone reductase that prevents the autoxidation of the labile intermediate 1,2,4-benzenetriol to 2-hydroxy-1,4-benzoquinone (Takenaka et al., 2011).

## Bacterial Degradation of Chloroaminophenols

**FIGURE 3:**
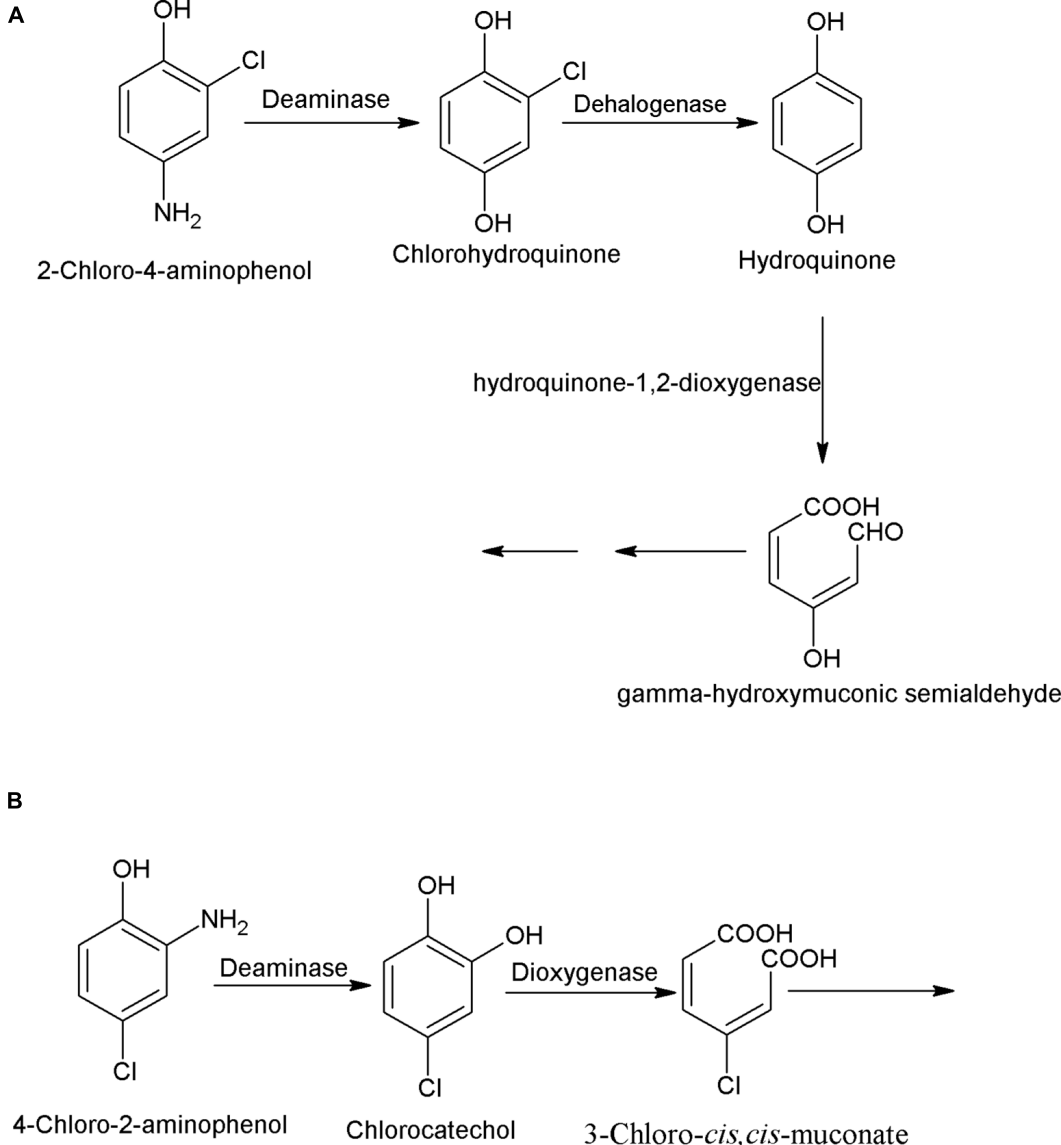
**Bacterial degradation pathway of **(A)** 2-chloro-4-aminophenol in *Arthrobacter* sp. SPG (Arora et al., 2014a) and **(B)** 4-chloro-2-aminophenol in *Burkholderia* sp. RKJ 800 (Arora et al., 2014b)**.

Chloroaminophenols (chlorinated derivatives of aminophenols) are widely used in dye synthesis. In this subsection, pathways for bacterial degradation of 2-chloro-4-aminophenol (2C4AP) and 4-chloro-2-aminophenol (4C2AP) are described. The degradation pathway of 2C4AP was studied in an *Arthrobacter* sp. SPG that utilized 2C4AP as its sole source of carbon and energy (Arora et al., 2014a). The initial step of the 2C4AP degradation is deaminase-catalyzed hydrolytic deamination of 2C4AP into chlorohydroquinone (CHQ; Arora et al., 2014a). The next step, catalyzed by a CHQ-dehalogenase involves reductive dehalogenation of CHQ to hydroquinone (HQ). Further degradation of HQ proceeds via ring cleavage, catalyzed by HQ- dioxygenase (**Figure 3A**).

Another bacterium, *Burkholderia* sp. RKJ 800, which utilizes 4C2AP as its sole carbon and energy, degrades it via chlorocatechol (Arora et al., 2014b). The 4C2AP degradation pathway is initiated with hydrolytic deamination of 4C2AP to 4-chlorocatechol by a 4C2AP-deaminase (Arora et al., 2014b). The next step, catalyzed by a 4-chlorocatechol-1,2-dioxygenase involves ring cleavage of 4-chlorocatechol into 3-chloro-*cis*,*cis*-muconate (**Figure 3B**).

## Bacterial Degradation of 2-Aminobenzoic Acid (Anthranilate)

Anthranilate is a key metabolite of bacterial degradation of several aromatic compounds, including tryptophan (Hayaishi and Stanier, 1951) indole (Fujioka and Wada, 1968), 4-chloroindole (Arora and Bae, 2015), 2-nitrobenzoate (Cain, 1966), quinaldine (Fetzner, 2000), and carbazole (Nojir et al., 2001). Several aerobic degradation pathways have been proposed for bacterial degradation of anthranilate and these pathways are the catechol pathway (Kim et al., 2015), the gentisate pathway (Cain, 1968), the 3-hydroxyanthranilate pathway (Liu et al., 2010) and the 2-aminobenzoyl-CoA pathway (Altenschmidt and Fuchs, 1992).

**FIGURE 4:**
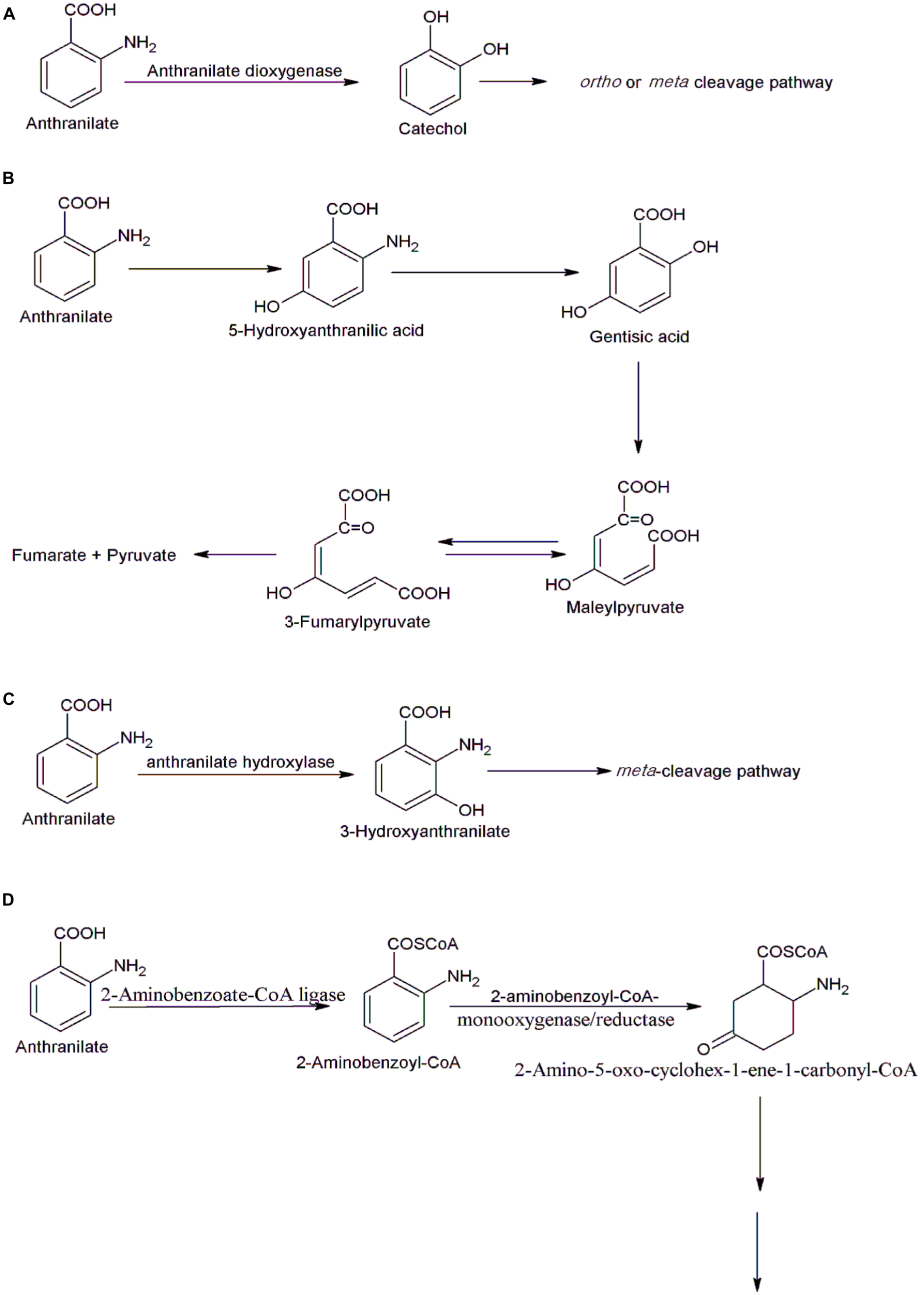
**Various bacterial degradation pathways of anthranilate. (A)** The catechol pathway in *Burkholderia cepacia* DBO1(Chang et al., 2003), *Acinetobacter* sp. ADP1 (Eby et al., 2001), *Pseudomonas aeruginosa* PAO1 (Costaglioli et al., 2012), and *Pseudomonas* sp. PAMC 2593 (Kim et al., 2015), **(B)** the gentisate pathway in *Nocardia opaca* (Cain, 1968), **(C)** the 3-hydroxyanthranilate pathway in *Geobacillus thermodenitrificans* NG80-2 (Liu et al., 2010), and **(D)** the 2-aminobenzoyl-CoA pathway in *Azoarcus evansii* (Schühle et al., 2001).

Most of the bacteria degrade anthranilate via the catechol pathway in which anthranilate-1,2-dioxygenase catalyzes conversion of anthranilate to catechol, which is degraded further via the *ortho-* or *meta*-cleavage pathway (Chang et al., 2003) (**Figure 4A**). The enzyme anthranilate-1,2-dioxygenase has been characterized from a number of bacteria (Eby et al., 2001; Chang et al., 2003). In *Burkholderia cepacia* DBO1, it is a three-component Rieske-type [2Fe-2S] dioxygenase with a reductase, a ferredoxin, and a two-subunit oxygenase (Chang et al., 2003). In *Acinetobacter* sp. ADP1 (Eby et al., 2001), *P. aeruginosa* PAO1 (Costaglioli et al., 2012) and *P. putida* P111, it is a two component complex composed of an oxygenase and a reductase. Kim et al. (2015) cloned and expressed the genes involved in the anthranilate degradation pathway from *Pseudomonas* sp. PAMC 2593. Two gene clusters have been identified in this strain; the *antABC* encodes the enzyme anthranilate dioxygenase that converts anthranilate to catechol whereas the *catBCA* encodes a catechol dioxygenase that cleaves to catechol to *cis*, *cis*–muconic acid (Kim et al., 2015).

*Nocardia opaca* degrades anthranilate via both catechol and gentisate (Cain, 1968). The gentisate pathway is a secondary route of anthranilate degradation in *N. opaca.* The gentisate pathway proceeds via formation of 5-hydroxyanthranilate, gentisate, maleylpyruvate, and pyruvate (Cain, 1968) (**Figure 4B**).

The 3-hydroxyanthranilate pathway of anthranilate degradation involves an anthranilate hydroxylase-catalyzed conversion of anthranilate to 3-hydroxyanthranilate that is further degraded via the *meta*-cleavage (Liu et al., 2010) (**Figure 4C**). The genes involved in this pathway have been identified and characterized in *Geobacillus thermodenitrificans* NG80-2 (Liu et al., 2010). The gene encoding anthranilate hydroxylase has been cloned, and expressed in *E. coli* and the purified protein was FAD-dependent hydroxylase. Two additional enzymes, riboflavin kinase/FMN adenylyltransferase and an FAD reductase, provide FAD for the anthranilate hydroxylase and genes encoding these enzymes were located in the same cluster in which gene encoding hydroxylase was located (Liu et al., 2010).

Another pathway of aerobic degradation of anthranilate was studied in *Azoarcus evansii* (Altenschmidt and Fuchs, 1992; Schühle et al., 2001). In the initial step, 2-aminobenzoate is activated to 2-aminobenzoyl-CoA by an AMP-forming 2-aminobenzoate-CoA ligase. 2-Aminobenzoyl-CoA is then transformed to a non-aromatic product, 2-amino-5-oxo-cyclohex-1-ene-1-carbonyl-CoA by a flavoenzyme, 2-aminobenzoyl-CoA monooxygenase/reductase (Schühle et al., 2001) (**Figure 4D**). Further degradation of 2-amino-5-oxo-cyclohex-1-ene-1-carbonyl-CoA occurs by the enzymes of β-oxidation (Schühle et al., 2001). The enzymes involved in the initial steps have been purified and characterized. An enzyme 2-aminobenzoate-CoA ligase is a monomeric protein of 65-kDa whereas 2-aminobenzoyl-CoA monooxygenase/reductase is homodimeric protein of 170 kd. The genes encoding enzymes of anthranilate degradation pathways were located on a small plasmid in *Azoarcus evansii* (Altenschmidt and Fuchs, 1992). Under anaerobic conditions, cells of *Azoarcus evansii* converted anthranilate to benzoyl CoA. This is two step reaction catalyzed by a 2-aminobenzoate-CoA ligase and 2-aminobenzoyl-CoA reductase (Schühle et al., 2001). The benzoyl CoA is degraded further via central CoA degradation pathway (Schühle et al., 2001).

## Bacterial Degradation of 5-Nitroanthranilate

**FIGURE 5:**
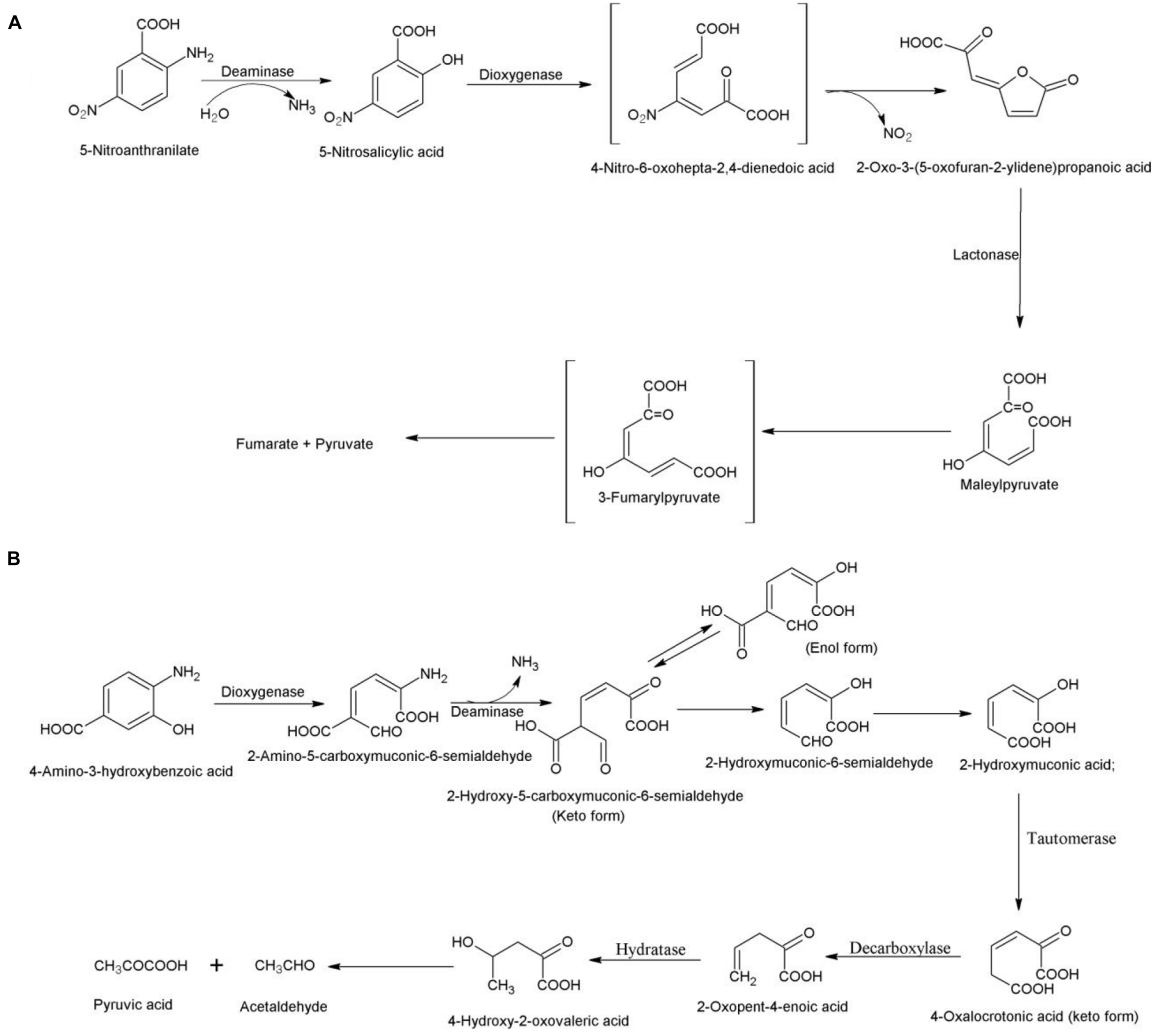
**Bacterial degradation pathway of **(A)** 5-nitroanthranilate in *Bradyrhizobium* sp. JS329 (Qu and Spain, 2010) and **(B)** 4-amino-3-hydroxybenzoate in *Bordetella* sp. 10d (Orii et al., 2006)**.

5-Nitroanthranilate is a natural nitroaniline that is produced by the soil bacterium *Streptomyces scabiei* (the predominant causal agent of common scab of potato in North America; Qu and Spain, 2010). The degradation of this compound was studied in *Bradyrhizobium* sp. JS329 that utilizes it as its sole source of carbon, nitrogen and energy (Qu and Spain, 2010). The degradation pathway is initiated with hydrolytic deamination of 5-nitroanthranilate to 5-nitrosalicylic acid by 5-nitroanthranilate deaminase. Second step involves 5-nitrosalicylic dioxygenase-catalyzed ring cleavage of 5-nitrosalicylic acid without prior removal of nitro group (Qu and Spain, 2011). The nitro group is eliminated either during the ring fission or immediately following it and the product undergoes spontaneous lactonization. In the next step, lactone is hydrolyzed to maleylpyruvate by a 2-oxo-3-(5-oxofuran-2-ylidene) propanoate lactonase (**Figure 5A**). The maleylpyruvate is further degraded via 3-fumarylpyruvate (Qu and Spain, 2011).

## Bacterial Degradation of 4-Amino-3-Hydroxybenzoate

The degradation pathway of 4-amino-3-hydroxybenzoate was studied in 4-amino-3-hydroxybenzoate-assimilating *Bordetella* sp. 10d (Orii et al., 2006). The degradation pathway is initiated with conversion of 4-amino-3-hydroxybenzoate to 2-amino-5-carboxymuconic-6-semialdehyde by a 2-amino-3-hydroxybenzoate-2,3-dioxygenase (Orii et al., 2006). The next step, catalyzed by 2-amino-5-carboxymuconic-6-semialdehyde deaminase involves deamination of 2-amino-5-carboxymuconic 6-semialdehyde to 5-carboxymuconic-6-semialdehyde that undergoes non-enzymatic carboxylation to form 2-hydroxymuconic-6-semialdehyde (**Figure 5B**). The hydroxymuconic-6-semialdehyde dehydrogenates to 2-hydroxymuconic acid that is converted to 4-oxalocrotonate by 4-oxalocrotonate tautomerase (Orii et al., 2006). In the next step, 4-oxalocrotonate decarboxylase catalyzes decorboxylation of 4-oxalocrotonate to 2-oxopent-4-enoic acid that is converted to 4-hydroxy-2-oxovaleric acid by a 2-oxopent-4-enoate hydratase. 4-Hydroxy-2-oxovaleric acid is metabolized to pyruvic acid and acetaldehyde (Orii et al., 2006). The enzymes, 2-amino-3-hydroxybenzoate-2,3-dioxygenase and 2-amino-5-carboxymuconic-6-semialdehyde deaminase have been cloned and characterized from *Bordetella* sp. 10d (Murakami et al., 2004; Takenaka et al., 2009).

## Bacterial Degradation of Methylanilines and Their Derivatives

In this subsection, the pathways for the bacterial degradation of methylanilines and their derivatives are summarized. *P. testosterone* can use 4-methylaniline (*p*-toluidine) as its sole source of carbon and energy and degraded it via 4-methylcatechol and 2-hydroxy-5-methyl-*cis,cis*-muconate semialdehyde (Raabe et al., 1984). The initial oxidation of *p*-toluidine resulted in formation of 4-methylcatechol that ring-cleaved to 2-hydroxy-5-methyl-*cis,cis*-muconate semialdehyde by a *meta*-pyrocatechase (Raabe et al., 1984). Anaerobic degradation of 4-methylaniline was studied in anaerobic sulfate-reducing bacterium, *Desulfobacula toluolica* Tol2 that transformed it into *p*-aminophenylacetic acid and phenylacetic acid as dead end products (Raber et al., 1998).

Another bacterium*, P. cepacia* strain CMA1 utilized 3-chloro-4-methylaniline as its sole source of carbon and energy and degraded it via liberation of ammonium and chloride (Stockinger et al., 1992). Authors anticipated that the initial step of degradation of 3-chloro-4-methylaniline in strain CMA1 is an aniline oxygenase-catalyzed reaction with possible formation of chloromethylcatechol that degraded further via an *ortho*-cleavage pathway (Stockinger et al., 1992). Fuchs et al. (1991) reported co-metabolism of 2-methylaniline, 4-chloro-2-methylaniline, and 3-chloro-2-methylaniline in the presence of ethanol as additional carbon source by two strains of *Rhodococcus rhodochrous*, wild-type strain CTM and its spontaneous mutant strain CTM-1 (Fuchs et al., 1991). Strain CTM degraded 2-methylaniline via 3-methylcatechol that degraded further via a *meta*-cleavage pathway (Fuchs et al., 1991). A spontaneous mutant strain CTM-1 lacking the enzyme of *meta*-cleavage pathway degraded 2-methylcatechol via the *ortho*-cleavage pathway (Fuchs et al., 1991). Strain CTM degraded 4-chloro-2-methylaniline via 5-chloro-3-methylcatechol that degraded further via the *ortho*-cleavage pathway (Fuchs et al., 1991). Strain CTM degraded 3-chloro-2-methylaniline via 4-chloro-3-methylcatechol that was further converted to 2-hydroxy-5-chloro-6-oxoheptanoic acid which was accumulated in media (Fuchs et al., 1991).

## Bacterial Degradation of Chloroanilines

Chloroanilines including monochloroanilines and dichloroanilines are chloro derivatives of aniline, which are widely used for in the industrial production of dyes, cosmetics, pharmaceutical products, and herbicides. In this subsection, the pathways for the bacterial degradation of monochloroanilines and dichloroanilines are summarized. Many bacteria have been isolated and characterized with their ability to degrade monochloroanilines (4-chloroaniline, 3-chloroaniline, and 2-chloroaniline). Examples are *Pseudomonas* sp. JL2, *Delftia acidovorans* CA28, *Comamonas testosteroni* 12, *Acinetobacter baumannii* CA2, *P. putida* CA16, *Delftia tsuruhatensis* H1, and *Acinetobacter baylyi* GFJ2 (Latorre et al., 1984; Loidl et al., 1990; Boon et al., 2001; Vangnai and Petchkroh, 2007; Zhang et al., 2010a; Hongsawat and Vangnai, 2011). The initial step of monochloroaniline degradation is an oxidative deamination of monochloroaniline to the corresponding chlorocatechol by aniline dioxygenase. The chlorocatechol is degraded further via either the *ortho*-cleavage pathway or the *meta*-cleavage pathway. Most of chloroaniline degrading bacteria degrade aniline via the *ortho*-cleavage pathway. *P. acidovorans* CA50 mineralized 2-chloroaniline via the modified *ortho*-cleavage pathway (Hinteregger et al., 1994). *Brevundimonas diminuta* INMI KS-7 also degrades 3-chloroaniline and 4-chloroaniline via the *ortho*-cleavage pathway with formation of 4-chloropyrocatechol, 3-chloromuconic acid, maleylacetic acid and 3-ketoadipic acid (Surovtseva et al., 1986). The aniline degradation via the *meta*-cleavage pathway has been observed in *Diaphorobacter* PCA039, which metabolizes 4-chloroaniline via 4-chlorocatechol, 2-hydroxy-5-chloromuconic semialdehyde, 5-chloro-4-oxalocrotonate, and 5-chloro-2-oxo-4-hydroxypentanoate (Zhang et al., 2010b). Similarly, *C. testosteroni* 12 metabolizes 3-chloroaniline via the *meta*-cleavage pathway (Boon et al., 2000). Król et al. (2012) showed that chloroaniline dioxygenase of *C. testosteroni* WDL7 is a multicomponent enzyme consisting of large and small subunits of dioxygenase (encoded by *dcaA1*, *dcaA2)*, and a reductase (encoded by *dcaA3*). The large and small subunits of chloroaniline dioxygenase of *Comamonas testosteroni* WDL7 shows significant amino acid sequence identity with aniline dioxygenase large and small subunits of aniline-utilizing bacteria, *Delftia acidovorans* 7N, *P. putida* UCC22, *Delftia tsuruhatensis* AD9, and *Frateuria* sp. ANA-18 (Fukumori and Saint, 1997; Murakami et al., 2003; Urata et al., 2004; Liang et al., 2005). Nitisakulkan et al. (2014) reported oxidation of chloroanilines by the *P. putida* T57 toluene dioxygenase. *E. coli* expressing the *P. putida* toluene dioxygenase gene complex (products of the *todC1C2BA* genes) catalyzes 1,2- and 2,3-dioxygenation of 4-chloroaniline to form 4-chlorocatechol and 2-amino-5-chlorophenol. 5-chloropyrogallol is also formed due to dioxygenation of 4-chlorocatechol.

**FIGURE 6:**
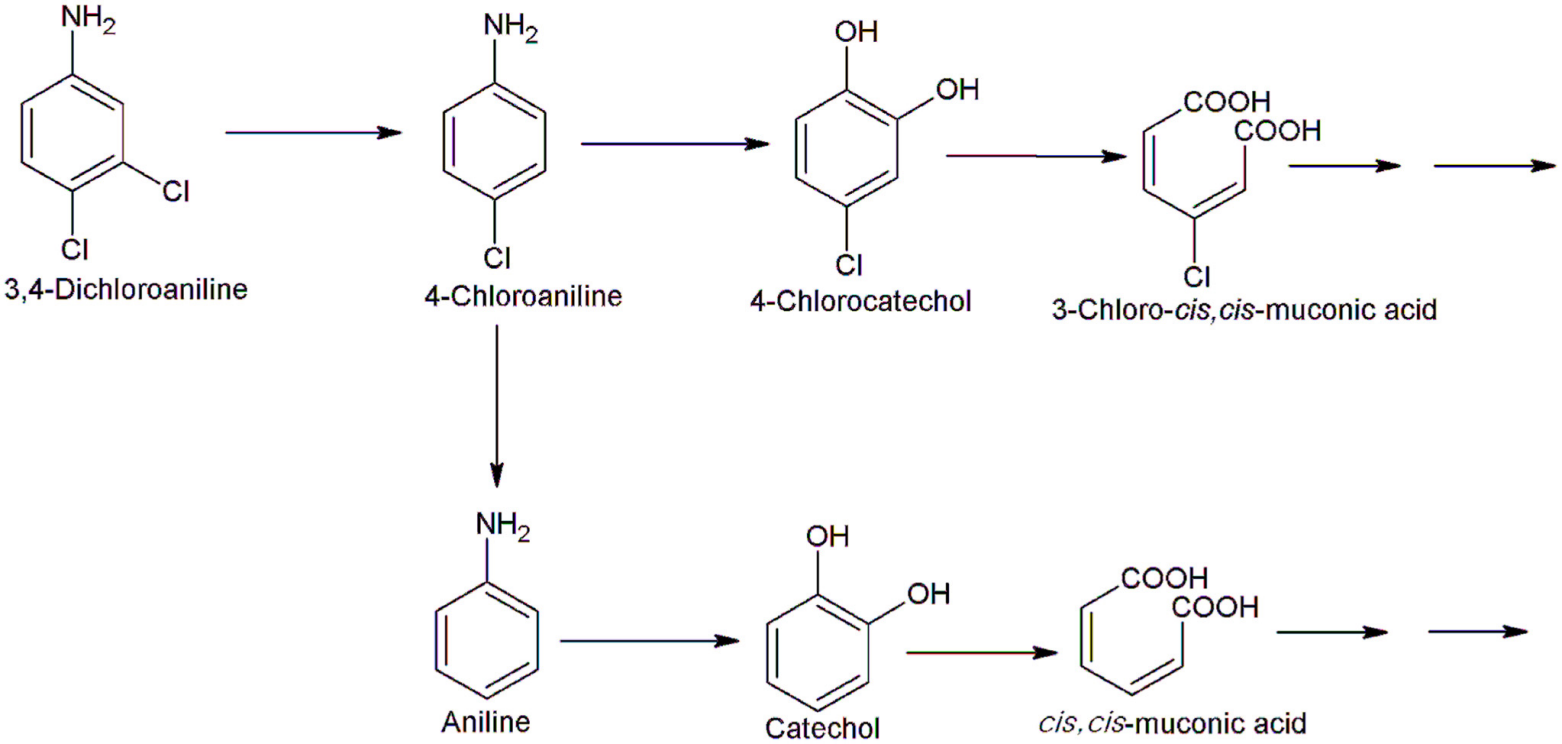
**Bacterial degradation pathway of 3,4-dichloroaniline in *Acinetobacter baylyi* GFJ2 (Hongsawat and Vangnai, 2011)**.

Bacterial degradation of dichloroanilines has also been investigated. *Bacillus megaterium* IMT21 and *Rhodococcus* sp. T1-1 utilized five isomers of dichloroaniline including 3,4-dichloroaniline, 2,3-dichloroaniline, 2,4-dichloroaniline, 2,5-dichloroaniline, and 3,5-dichloroanilines as their sole source of carbon and energy (Lee et al., 2008; Yao et al., 2011). Strain IMT21 degrades 2,3-dichloroaniline, 2,4-dichloroaniline and 2,5-dichloroaniline via dichloroaminophenol, and 3,4- and 3,5-dichloroaniline via dichloroacetanilide (Yao et al., 2011). However, no metabolite was detected in degradation of any of the dichloroaniline isomers by strain T1-1 (Lee et al., 2008). Another bacterium, *Alcaligenes faecalis* H1 mineralized 3,4-dichloroaniline via an initial oxidative deamination with formation of 4,5-dichloropyrocatechol (Surovtseva et al., 1993) whereas *P. fluorescens* 26-K mineralized 3,4-dichloroaniline via 4-amino-2-chlorophenol through initial dehalogenation and subsequent hydroxylation (Travkin et al., 2003). *Brevundimonas diminuta* INMI KS-7 degrades 3,4-dichloroaniline via 4,5-dichloropyrocatechol and dichloromuconic acid (Surovtseva et al., 1986). A strain of *Pseudomonas* sp. degrades 3.4-dichloroaniline in the presence of aniline via 4,5-dichlorocatechol, 3,4-dichloromuconate, 3-chlorobutenolide, 3-chloromaleylacetate, and 3-chloro-4-ketoadipate (You and Bartha, 1982). Branching of degradation pathway of 3,4-dichloroaniline was observed in *Acinetobacter baylyi* strain GFJ2 that utilized it as its sole source of carbon energy (Hongsawat and Vangnai, 2011). The initial step of degradation involves dehalogenation of 3,4-dichloroaniline to 4-chloroaniline which is further degraded via two different routes (**Figure 6**). In first route, 4-chloroaniline undergoes dehalogenation to produce aniline, which is further degraded via catechol and the *ortho*-cleavage pathway. In second route, 4-chloroaniline undergoes dioxygenation to 4-chlorocatechol and ammonia. Further degradation of 4-chlorocatechol proceeds via the *ortho*-ring cleavage pathway (Hongsawat and Vangnai, 2011).

Anaerobic degradation of 3,4-dichloroaniline was observed in a *Rhodococcus* sp. 2 that degraded it through reductive deamination to form 1,2-dichlorobenzene (Travkin et al., 2002). Other metabolites (3,4-dichloroacetanilide and 3,4-dichloro-*N*-(3,4-dichlorophenyl) benzamide) were also detected as transformation products of this anaerobic reaction (Travkin et al., 2002).

## Conclusion

Bacterial degradation of aniline and its chloro and methyl derivatives generally occurs via formation of corresponding catechols that degrade further via either the *ortho*-cleavage or the *meta*-cleavage pathway. The mechanism of catechol formation in aniline degradation has recently been postulated and the related genes and enzymes have been well-characterized. Future works on proteomics may increase our understanding towards bacterial degradation of aniline.

Bacterial degradation pathways for aminophenols and chloroaminophenols have also been studied. The degradation of these compounds generally initiated via either the ring cleavage or the hydrolytic deamination. The genes and enzymes involved in the aminophenol degradation have also been characterized whereas the genomics of the degradation pathways of chloroaminophenols have yet not studied.

Diverse mechanisms of the anthranilate degradation have been reported and four aerobic metabolic pathways including the catechol pathway, the gentisate pathway, the 3-hydroxyanthranilate pathway, and the 2-aminobenzoyl-CoA pathway have been proposed. Amongst, the catechol pathway is the most common route for anthranilate degradation.

Little is known about bacterial degradation of other monocyclic aromatic amines. More bacteria should be isolated by the enrichment method using monocyclic aromatic amines as substrates and the biochemical and molecular characterization of biodegradation of monocyclic aromatic amines should be carried out in these bacteria.

## Author Contributions

PA collected all the relevant publications, arranged the general structure of the review, drafted the text, and produced figures.

## Conflict of Interest Statement

The author declares that the research was conducted in the absence of any commercial or financial relationships that could be construed as a potential conflict of interest.
